# Developing a Consensus for Adolescent and Young Adult mHealth HIV Prevention Interventions in the United States: A Delphi Method Study

**DOI:** 10.2196/25982

**Published:** 2022-07-12

**Authors:** Kayla Knowles, Nadia Dowshen, Susan Lee, Amanda Tanner

**Affiliations:** 1 PolicyLab Children's Hospital of Philadelphia Roberts Center for Pediatric Research Philadelphia, PA United States; 2 Craig-Dalsimer Division of Adolescent Medicine Children's Hospital of Philadelphia Philadelphia, PA United States; 3 Perelman School of Medicine University of Pennsylvania Philadelphia, PA United States; 4 Department of Public Health Education University of North Carolina Greensboro Greensboro, NC United States

**Keywords:** HIV prevention and care, adolescents, mHealth, mobile apps, Delphi method, health application, HIV care, HIV prevention, health intervention, public health, health care, health accessibility, health technology, digital health, HIV

## Abstract

**Background:**

Engaging adolescents and young adults (AYAs) who are at elevated risk for HIV acquisition or who are living with HIV in health care has posed a major challenge in HIV prevention and care efforts. Mobile health (mHealth) interventions are a popular and accessible strategy to support AYA engagement despite barriers to care present along the HIV care continuum. Even with progress in the field of mHealth research, expert recommendations for the process of designing, evaluating, and implementing HIV-related mHealth interventions are underdeveloped.

**Objective:**

The aim of this study was to compile expert recommendations on the development, evaluation, and implementation of AYA-focused HIV prevention and care mHealth interventions.

**Methods:**

Experts from adolescent mHealth HIV research networks and investigators of recently funded HIV mHealth projects and programs were identified and invited to complete a series of electronic surveys related to the design, implementation, and evaluation of HIV-related mHealth interventions. A modified Delphi method was used to ask experts to score 35 survey items on a 4-point Likert scale from not important to very important and encouraged experts to leave additional comments in textboxes. Responses were reviewed by the researchers, a team of 4 HIV mHealth intervention experts. The average importance ratings from survey responses were calculated and then categorized as retained (high importance), flagged (mid-level importance), or dropped (no/low importance). Additionally, thematic analysis of expert comments helped modify survey items for the next survey round. An evaluation of the level of agreement among experts on the most important items followed each round until consensus was reached.

**Results:**

Of the 35 invited experts, 23 completed the first survey representing a variety of roles within a research team. Following two rounds of Delphi surveys, experts scored 24 of the 28 (86%) survey items included in round two as important to very important. The final consensus items included 24 recommendations related to the mHealth intervention design process (n=15), evaluation (n=2), and implementation (n=7). The 3 survey items with the highest average scores focused on the design process, specifically, (1) the creation of a diverse team including researchers, app software developers, and youth representation; (2) the importance of AYA-focused content; and (3) the value of an iterative process. Additionally, experts highlighted the importance of establishing the best ways to collect data and the types of data for collection during the evaluation process as well as constructing a plan for participant technology disruption when implementing an mHealth intervention.

**Conclusions:**

The modified Delphi method was a useful tool to convene experts to determine recommendations for AYA-focused HIV prevention and care mHealth interventions. These recommendations can inform future mHealth interventions. To ensure the acceptability, feasibility, and efficacy of these AYA HIV prevention interventions, the focus must be on the specific needs of AYAs by including representation of AYAs in the process, including consistent and relevant content, ensuring appropriate data is collected, and considering technology and health accessibility barriers.

## Introduction

In the United States, HIV is a major cause of morbidity, mortality, and social adversity among adolescents and young adults (AYAs) [1]. In 2019, individuals aged 13-24 years accounted for 21% of newly reported HIV infections [2]. AYAs have poor health outcomes across the HIV care continuum—diagnosis, linkage and retention in care, maintaining an antiretroviral therapy (ART) regimen, and achieving viral suppression [2]. Over 40% of youth living with HIV (YLH) in the United States are unaware of their status compared to 14% of adults [3]. Additionally, for YLH with a known HIV diagnosis, care engagement was greater than the overall population with HIV diagnosis, yet viral suppression was 60% [3], again fairing worse than their adult counterparts. Beyond age, disparities based on race, gender, and sexual orientation have created an environment where communities with the highest incidence of HIV infections have access to the fewest resources [2-4]. Young Black and Latinx gay, bisexual, other men who have sex with men (GBMSM), and transgender women are at the greatest risk of becoming infected with HIV in their lifetime [3]. Experiences of pervasive stigma and systemic racism in and outside health care systems prevent communities most vulnerable to HIV from accessing and engaging in the HIV care continuum [3,5]. High rates of sexual risk behaviors, low rates of HIV testing, and poor engagement in HIV care [2,3] have generated a high demand for culturally competent and accessible AYA-targeted HIV prevention and treatment interventions.

The US Department of Health and Human Services Ending the HIV Epidemic (EHE) initiative outlined strategies to reduce new HIV infections by 90% by 2030 [6]. Efficient implementation of HIV research into practice was an area of focus included in the National HIV/AIDS Strategy to move toward EHE, as well as prioritizing populations most affected by the HIV epidemic [7]. In 2018, more than 90% of US youth regardless of race, income, or parent’s education level had access to a smartphone [8], and adolescents expressed a preference for HIV interventions on mobile technologies [9,10]. Hence, mobile health (mHealth) approaches have become increasingly common in engaging AYAs in HIV prevention and care efforts. Focus groups and usability testing studies with YLH have identified privacy, entertainment, social support, and educational content as desired features of mHealth HIV interventions [11-14]. To date, mHealth interventions have used a variety of app features to support engagement, including connecting with other YLH via in-app forums or message boards [15], daily or weekly SMS text message reminders [16-18], and educational games [10,19]. Many mHealth projects, such as weCare, MyPEEPS, and Guy2Guy, prioritize engagement of GBMSM and transgender women with a social support network, inclusive sex education, and culturally relevant content [15,20,21]. mHealth interventions have shown some improvement in outcomes of interest including ART adherence, viral suppression, medical appointment attendance, HIV testing, and perceived social support [10,15,19,20,22]. However, few of these interventions have been tested in large-scale randomized controlled trials, and fewer have been implemented into routine clinical care or community-based settings. Further, there are no existing recommendations for developing, testing, and disseminating mHealth HIV prevention or treatment interventions for AYAs. Accordingly, this study aimed to use a modified Delphi method [23,24] to elicit expert recommendations on the design, evaluation, and implementation of AYA-focused HIV prevention and care mHealth interventions. Study design refers to the road map used to collect and analyze data based on a specific question the researchers hope to answer [25]. An evaluation reflects on how the intervention is implemented and how effective it is over time [26]. An effective evaluation will allow an intervention to adapt over time. Finally, implementation is the adaption and delivery of evidence-based research findings into real-world practice to benefit the community that the intervention was developed to benefit [27].

## Methods

### Overview

The Delphi method is based on the convergence of expert opinion after multiple rounds of data collection (eg, surveys with Likert scale items) with the final round occurring when agreement emerges among experts [23]. The multiple rounds of data collection and analysis in the Delphi method allow for elimination of survey items that experts agree are unimportant and refinement of the remaining items [23]. The final round leaves a consensus list of recommendations experts agree are crucial to consider. This study used a modified Delphi method [24] to reach consensus on expert recommendations for AYA HIV mHealth interventions. The modified Delphi method used in this study maintained the multiple rounds of data collection, but it omitted the expert-to-expert face-to-face discussion that would occur following data collection due to difficulties in varying schedules and time zones as well as to limit experts from influencing each other. In the absence of the expert discussion, participants had the option to leave text comments in the survey that were later considered when designing the next survey data collection round.

### Study Design

#### Survey Development

Survey items were generated by 4 mHealth HIV experts, who were investigators for various federally funded projects relevant to this topic. A list of common considerations when developing, evaluating, and implementing HIV mHealth interventions were compiled for the survey. The study team and the survey development group reviewed and edited the initial survey to ensure items were concise and minimized influence on expert participants’ scoring.

The consensus survey for the first round included 35 Likert items sorted into five different categories: (1) design: preparation phase; (2) design: core elements; (3) evaluation: preparation and study design; (4) evaluation: data sources; and (5) implementation. Item response options ranged from not important (1) to very important (4), and each item included a “comment” textbox for experts to type in general comments (eg, why they scored an item the way they did) or revised wording suggestions. The survey was programmed in Research Electronic Data Capture (REDCap), a secure website software used for data collection and management in clinical and translational research [28,29], which was used to disseminate the survey to expert participants.

#### Expert Identification and Selection

The identification of experts who have knowledge of and experience with AYA mHealth HIV prevention and care interventions to participate in this study involved a review of large-scale HIV mHealth intervention networks and recently funded HIV mHealth projects and programs at the start of this study in 2019. Expert participants were identified through two different adolescent mHealth HIV-related networks, the iTech U19 of the National Institutes of Health (NIH)–funded Adolescent Medicine Trials Network [30] and the Health Resources and Services Administration’s Special Projects of National Significance Initiative in Use of Social Media to Improve Engagement, Retention, and Health Outcomes along the HIV Care Continuum, 2015-2019 [31]. Additional experts were identified through a search of 2019 grantees of adolescent HIV mHealth interventions in the NIH Research Portfolio Online Reporting Tools Expenditures and Results [32] with keyword Medical Subject Headings (MeSH) [33] terms “adolescent,” “HIV,” and “mobile health.” Expert identification and selection for survey participation aimed to encompass the involvement of a variety of cities and organizations in the United States and to include representation of principal investigators, program directors, project coordinators, protocol chairs, and project staff to capture the range of perspectives and expertise within a study team.

#### Survey Distribution and Data Collection

The survey was distributed via email with a weblink to participate in the REDCap survey. Experts had a 4-week window to complete the survey. After the round 1 survey window closed, the data were deidentified and analyzed. Survey items were modified based on expert participant survey response scores and accompanying comments resulting in a subsequent round 2 survey. Given the number of invited experts and consideration of their daily responsibilities, instead of having experts review each other’s responses in their entirety, experts were informed of the group’s overall responses by presenting them only with retained and reworded survey items in the next round for efficiency and privacy. A subsequent round was conducted similarly until a consensus was reached by participants. Only participants from the first round were eligible to participate in the subsequent round. Ultimately, the process was completed in two survey rounds between November 2019 and April 2020. Following successful completion of participation, expert participants were compensated in the form of a US $50 e–gift card for their time and effort in this study.

### Data Analysis and Consensus Criteria

Deidentified survey Likert responses were exported from REDCap for analysis in Excel (Microsoft Corporation). Scores for each survey responses were determined based on the mean importance rating from not important (1) to very important (4) and were then categorized as retained, flagged, or dropped. Retained survey responses were those with mean scores between 3 (important) and 4 (very important), and automatically appeared on the next iteration of the survey. Flagged survey responses had a score between 2 (somewhat important) and 3 (important). For flagged survey responses, the SDs were calculated. The survey responses with an SD>1 were dropped, and the other flagged survey responses were revised based on the experts’ comments. Finally, survey responses with a score ≤2 (not at all important, 1, to somewhat important, 2) were dropped.

As part of the REDCap survey, expert comments were collected in textboxes, which provided additional information on why they chose a particular survey response, and were reviewed during analysis [34]. Comments were compiled in Word (Microsoft Word) under their survey item, and responses with similar explanations/questions were grouped together for review. Comments were used to inform revisions following round 1 and were especially helpful for reviewing the flagged items, considering where divergence reflected disagreement among experts or unclear wording. Comments were also used to assist in assessing consensus among experts. The revised recommendation list presented to experts were those assessed as “important” (3) to “very important” (4) following the round 2 survey.

### Ethical Considerations

This study received an exempt determination from the Children’s Hospital of Philadelphia’s Institutional Review Board. The beginning of each survey included an overview of the study and consent language where moving to the next page of the survey indicated willingness to participate in the study.

## Results

### Expert Participation

**Figure 1:**
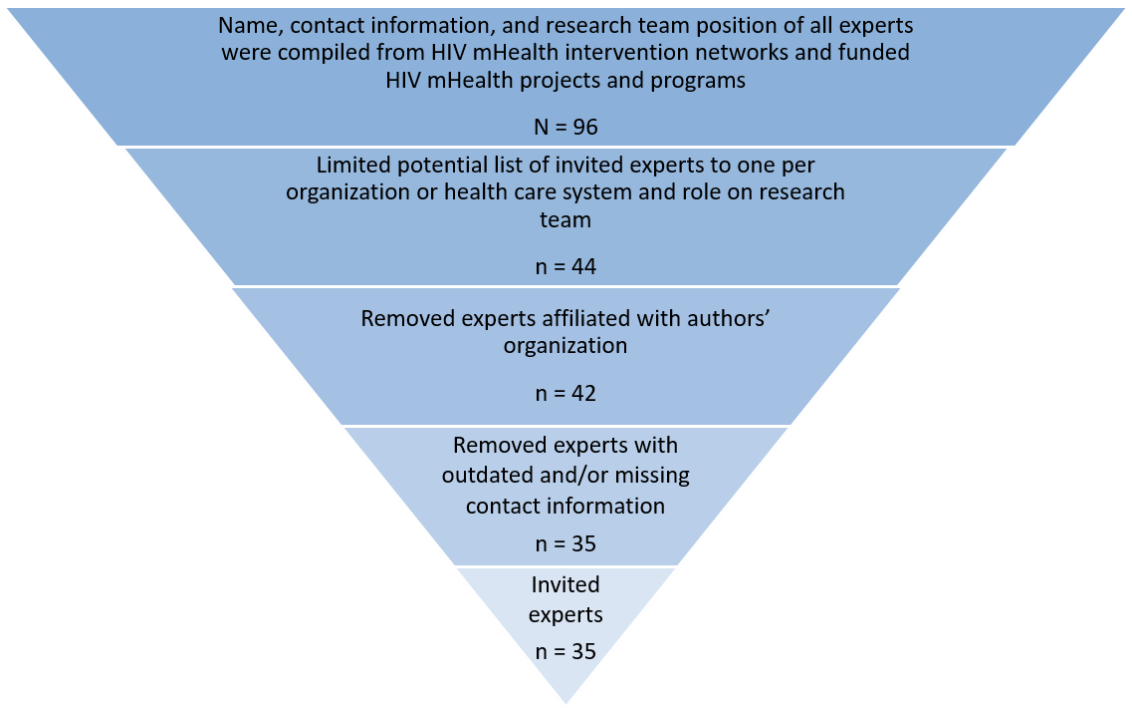
Expert identification and selection.

Survey responses were received from 23 of the 35 (66%) invited adolescent HIV/mHealth experts for round 1 (see Figure 1) identified using reports from HIV mHealth intervention networks and recently funded HIV mHealth projects and programs. Participating experts represented 20 research organizations across the United States. More than half were principal investigators (n=15) and the remaining experts included various roles on a study team, including project directors (n=3), evaluators (n=2), project coordinators (n=2), and other project staff (n=1). Experts who completed the first survey were reinvited to participate in round 2 with a high response rate of 21 of 23 (91%) experts.

### Round 1

#### Item Score Analysis

Of the 35 items asked in the round 1 survey, 86% (n=30) fit the criteria to be retained, 14% (n=5) were identified as flagged, and none fit the dropped criteria (not important, 1, to somewhat important, 2) following the analysis of the survey responses. Table 1 displays the expert responses for each section: total retained, flagged by SD, and dropped item. Of the 5 flagged items, 1 had an SD>1 and subsequently was kept for round 2.

**Table 1:** Consensus survey response scoring results: round 1 (R1; n=35) and round 2 (R2; n=28).

					Design: preparation			Design: core intervention elements			Evaluation: preparation and study design			Evaluation: data source			Evaluation: preparation, study design, and data source			Implementation			Total, n
**Total, n**																							
	R1			13			7			4			4			N/A^a^			7			35	
	R2			11			7			N/A			N/A			3			7			28	
**Mean (SD)**																							
	R1			3.34 (0.39)			3.60 (0.28)			3.17 (0.28)			3.19 (0.20)			N/A			3.43 (0.20)			N/A	
	R2			3.41 (0.35)			3.53 (0.31)			N/A			N/A			3.11 (0.32)			3.41 (0.17)			N/A	
**Retain, n**																							
	R1			10			7			3			3			N/A			7			30	
	R2			9			6			N/A			N/A			2			7			24	
**Flag, n^b^**																							
	**R1**																						
		SD>1	1			0			0			0			N/A			0			1		
		SD≤1	2			0			1			1			N/A			0			4		
	**R2**																						
		SD>1	0			0			N/A			N/A			0			0			0		
		SD≤1	2			1			N/A			N/A			1			0			4		
**Drop, n**																							
	R1			0			0			0			0			N/A			0			0	
	R2			0			0			N/A			N/A			0			0			0	

^a^N/A: not applicable.

^b^Flagged items with SD≤1 were dropped.

#### Experts’ Supplemental Comments

Themes emerged within the comment section that guided the revision of the round 2 survey. Various questions by experts reflected a misunderstanding about how to rate an item’s importance (eg, based on their specific projects or mHealth HIV interventions generally). For round 2, a clarifying statement was included in the instructions to correct this issue. Experts were reminded to focus on overall recommendations that are important even if they do not apply to all intervention types. Additionally, some items (n=6) were revised based on feedback that they were unclear and required further clarification.

#### Retained Items

After review of the quantitative and qualitative analyses, decisions were made about which items to retain for the next round. A total of 4 flagged items with a SD≤1 were dropped, and 3 others were eliminated due to feedback related to the similarities among items in the evaluation: data source section. Specific data sources such as paradata, electronic medical records (EMRs), self-reports, and qualitative data individually were found to be less important than generally identifying an appropriate data source for evaluation of mHealth HIV interventions. Therefore, 3 data source questions were dropped and 1 was revised into a single simplified data source item. The round 2 survey included 28 survey items reduced from the original 35 survey items in the round 1 survey.

### Round 2

#### Item Score Analysis

Experts were asked to score the importance of the revised 28-item survey. Following the same analytical process as in round 1 (see Table 1), 24 survey items were retained, 4 were flagged, and none fit the dropped criteria (see Table 1). The 4 items that scored in the flagged range (somewhat important, 2, and important, 3) in both round 1 and again in round 2 (see Table 2) were subsequently removed from the recommendation list. Over the two rounds of data collection, experts scored these flagged items consistently as less important compared to the retained items (important, 3, and very important, 4).

**Table 2:** Rounds 1 and 2 scores for flagged items that were dropped from expert’s recommendation list.

Flagged items		Round 1, mean (SD)	Round 2, mean (SD)
**Design: preparation**			
	Weigh the advantages and disadvantages of integration with an electronic medical record	2.83 (0.94)	2.81 (0.81)
	Consider the advantages and disadvantages of an intervention that is publicly available vs offered by providers in a clinical setting	3.09 (0.73)	2.86 (1.06)
**Design: core intervention elements**			
	Use a theoretical foundation to guide intervention development	3.00 (0.85)	2.95 (0.74)
**Evaluation: preparation, study design, and data source**			
	Consider a study design other than traditional randomized controlled trials, such as a multiphase optimization strategy, sequential multiple assignment randomized trials, or pragmatic trials to optimize and tailor the intervention	3.13 (0.82)	2.76 (0.70)

#### Expert Recommendations

Overall, 86% of the items in round 2 were scored by experts as important (3) to very important (4), indicating a consensus was reached, and a third round of survey data collection was not needed. Table 3 provides the experts’ final 24 recommendations for developing, evaluating, and implementing HIV prevention and care mHealth interventions.

As shown in Table 3, the majority (15/24) of the final recommendations experts assessed as the most important were related to intervention design. Design encompasses a broad range of considerations from who will be on the design team, cost, content, privacy concerns, and more. For example, expert made the following comments on the importance of design consideration:

...The intervention is not for us. It is not something fun to do with hundreds of thousands of dollars and hours. It is a product we're building with our population to make impactful and positive change in the lives and health of our target population.

On AYA-specific content

Having the intervention be stealth during everyday life is important. However, message content, when participants are interacting with the intervention, may need to push the privacy boundaries to make it relevant.

On importance of privacy

When it comes to youth (and perhaps people in general), novelty is important as is continuous development. Nothing is perfect from the beginning. There will always be something that can be tweaked, added, or removed in order to better serve the users of the technology

On incorporating an iterative process

Evaluation items made up 2 of the final recommendations. As discussed previously, data sources individually were not viewed as important as tailoring the evaluation to each intervention appropriately. Finally, there were 7 final implementation recommendations.

**Table 3:** Final expert recommendations and average importance scores (range 1-4: not important to very important).

			Round 1, mean (SD)		Round 2, mean (SD)
**Design: preparation recommendations**					
	1. Have a multidisciplinary team including experts in HIV, adolescent development, software programming, user interface design, and youth	3.83 (0.39)		3.81 (0.40)	
	2. Include youth throughout the process to maximize engagement	3.78 (0.52)		3.71 (0.56)	
	3. Weigh challenges and benefits of using existing technology (platforms or apps) vs newly created technology	3.30 (0.70)		3.24 (0.83)	
	4. Use an iterative process (incorporating feedback and refining) in developing or adapting the intervention	3.65 (0.49)		3.81 (0.51)	
	5. Design with sustainability in mind	3.52 (0.79)		3.52 (0.68)	
	6. Consider the balance between cost and functionality	3.56 (0.59)		3.38 (0.59)	
	7. Develop an intervention that is accessible, for instance it is platform agnostic (ie, can be used on Android, iOS, or Windows) and available to the majority of youth (ie, those with limited cell phone plans and access to Wi-Fi)	3.74 (0.54)		3.67 (0.58)	
	8. Determine the appropriate level of real human engagement (eg, automated messaging vs live human coach)	3.39 (0.84)		3.38 (0.59)	
	9. Consider whether you want to design for youth at various points across the continuum or for a specific target audience	3.36 (1.01)		3.33 (0.66)	
**Design: core intervention elements recommendations**					
	1. Ensure that intervention content is relevant to the needs of your specific youth population	3.74 (0.54)		3.95 (0.22)	
	2. Consider the appropriate dose/frequency of the intervention for optimal efficacy	3.61 (0.66)		3.57 (0.60)	
	3. Determine the most appropriate digital health modalities (eg, SMS text messaging, social media, mobile website, e-coach, app, or telemedicine) or a combination that will allow for the maximal engagement and effectiveness of intervention	3.74 (0.54)		3.52 (0.51)	
	4. Consider privacy and confidentiality in design (app icon, message content, home screen)	3.78 (0.52)		3.71 (0.46)	
	5. Ensure content and engagement strategies are developmentally and culturally appropriate	3.78 (0.42)		3.62 (0.50)	
	6. Maximize engagement strategies with the intervention to address issues with attrition	3.57 (0.51)		3.38 (0.67)	
**Evaluation: preparation, study design, and data source recommendations**					
	1. Determine which intervention components are most effective across groups or individuals	3.35 (0.78)		3.19 (0.68)	
	2. Establish the best ways to collect data and what types of data (eg, Google analytics, paradata, self-report data, or electronic medical record)	3.35 (0.65)		3.38 (0.67)	
**Implementation recommendations**					
	1. Consider the cost and logistics of any human component	3.52 (0.67)		3.38 (0.67)	
	2. Plan for participant technology disruption (lost, stolen, broken phone, or plan cut off) by collecting multiple modes of contact information and making it easy to reupload or log in to a platform	3.78 (0.52)		3.52 (0.51)	
	3. Consider integration into routine clinical care and community-based services where appropriate	3.17 (0.83)		3.48 (0.75)	
	4. Seek youth input about strategies to improve engagement with the intervention	3.43 (0.67)		3.62 (0.59)	
	5. Plan for how intervention informational content will be updated	3.48 (0.67)		3.33 (0.66)	
	6. Anticipate changes in platforms or operating systems	3.26 (0.69)		3.43 (0.68)	
	7. Consider strategies to meet milestones of potentially different timelines of research (eg, grant) and technology partners (eg, development and revision)	3.35 (0.83)		3.10 (0.70)	

## Discussion

### Principle Findings and Comparison With Prior Work

The modified Delphi method allowed for the rapid collection of data including the opinions of more than 20 AYA-focused mHealth HIV intervention experts with varied experience across the United States. Following two rounds of data collection, an original list of 35 items were modified into 24 recommendations on the design, evaluation, and implementation of HIV prevention and care mHealth interventions for AYAs. These consensus items provide guidelines for future development, evaluation, and implementation of mHealth interventions as well as for prevention scientists to support the health of AYAs at risk for acquiring or living with HIV. At each stage of AYA-focused mHealth HIV research, these considerations can guide decision-making to optimize efficacy, acceptability, and accessibility of mHealth technology to work toward ending the HIV epidemic.

The three items that experts scored as most important were related to intervention design: ensuring that intervention content is relevant to the needs of the specific youth population; having a multidisciplinary team including experts in HIV, adolescent development, software programming, user interface design, and youth; and using an iterative process (incorporating feedback and refining) in developing or adapting the intervention. These design recommendations highlight the potential value of youth involvement and using community-engaged research principles (eg, listening to your community stakeholders throughout the research process to build trust and improve health outcomes that outlast the study duration) [35,36]. These processes can ensure a strong foundation for the intervention as well as for intervention engagement and sustainability.

This study also identified that intrinsic elements of the design are critical for accessibility and privacy. The accessibility of mHealth interventions may fluctuate depending on the type of platform (eg, iOS, Android, or web applications), device’s operating system version, and adolescent’s access to Wi-Fi and data plan. Privacy and confidentiality of HIV-related mHealth interventions has been documented as very important to YLH [13,37,38], and AYA HIV experts in this study agreed. Preventing unwanted disclosures in fear of the stigma associated with HIV may stop adolescents from downloading or using mHealth interventions that do not protect their privacy and confidentiality [37]. Password-protected accounts, discrete app names, and limiting the access to one’s health information are ways to improve privacy and confidentiality [37,38].

The key considerations for the evaluation of mHealth interventions identified included determining what intervention components were the most effective and advantageous data collection strategies. The round 1 survey analysis found experts scored recommendations on individual data collection methods such as self-report, paradata, and EMRs as less important than selecting the right one for a specific project. This suggests that a one-size-fits-all approach to mHealth intervention evaluations may not be appropriate and should rather be tailored to fit each project’s goals and evolving technology. A clear understanding of the intervention’s desired outcomes may help to determine the most appropriate data collection methods.

In regard to real-world implementation of interventions, experts scored seeking youth input about strategies to improve engagement highest, indicating youth input would be the most important factor. Using a multidisciplinary team, including AYA input, from the start of intervention development was regarded as most important among experts. Again, the importance of including AYA input throughout the entirety of the project was recognized as crucial to reach and engage the youth audience the intervention was designed to help. Other highlights from the implementation recommendations primary focus on technology limitations such as high costs and frequent updates to platforms, operating systems, or devices. Understanding and taking into account the challenges facing youth most affected by HIV may help plan for a more successful implementation. For example, multiple forms of contact information, like emails or social media handles, may be helpful to collect since mobile phone disruptions are common in this group [39]. Additionally, staff time for technology maintenance and initiation were highlighted as important aspects to consider for intervention implementation for clinical care teams and community health organizations.

### Limitations

While not hosting a face-to-face discussion among experts allowed us to avoid scheduling challenges and reduced participant time burden, it remains a potential limitation to this study [23]. The option for experts to leave comments served as a discussion forum that allowed us to receive every expert’s opinion without influence from other experts. Another limitation was the potential for bias among experts invited to participate. Additionally, demographic, employment responsibilities, or years of experience were not collected. For this reason, researchers who occupied diverse roles on research teams were intentionally invited as experts to reduce this bias and gain a more complete understanding of different perspectives of the research design, evaluation, and implementation process.

### Conclusions

This study is among the first to propose expert recommendations on the development, evaluation, and implementation of mHealth interventions for HIV prevention among AYAs. With a clear focus on the role of youth in all aspects of the process, these expert opinions may not only help move forward the quality of technology-based research for adolescent HIV prevention but also ensure that successful interventions will be disseminated broadly for the most impact.

## Abbreviations

ART: antiretroviral therapy
AYA: adolescent and young adult
EHE: Ending the HIV Epidemic
EMR: electronic medical record
GBMSM: gay, bisexual, other men who have sex with men
MeSH: Medical Subject Headings
mHealth: mobile health
NIH: National Institutes of Health
REDCap: Research Electronic Data Capture
YLH: youth living with HIV
